# Synthesized economic evidence on the cost-effectiveness of screening familial hypercholesterolemia

**DOI:** 10.1186/s41256-024-00382-x

**Published:** 2024-09-26

**Authors:** Mengying Wang, Shan Jiang, Boyang Li, Bonny Parkinson, Jiao Lu, Kai Tan, Yuanyuan Gu, Shunping Li

**Affiliations:** 1 School of Management, Shanxi Medical University, Taiyuan, Shanxi China; 2 Macquarie Business School and Australian Institute of Health Innovation, Macquarie University Centre for the Health Economy, Macquarie University, Level 5, 75 Talavera Road, Macquarie Park, Sydney, NSW 2109 Australia; 3 School of Political Science and Public Administration, Wuhan University, Wuhan, Hubei China; 4 School of Public Policy and Administration, Xi’an Jiaotong University, Xi’an, Shaanxi, China; 5 Centre for Health Management and Policy Research, School of Public Health, Cheeloo College of Medicine, Shandong University, Jinan, Shandong China; 6 NHC Key Lab of Health Economics and Policy Research, Shandong University, Jinan, Shandong China; 7 Center for Health Preference Research, Shandong University, Jinan, Shandong China

**Keywords:** Health economics, Cost-effectiveness, Equity, Systematic review, Familial hypercholesterolemia, Screening

## Abstract

**Background:**

Familial hypercholesterolemia (FH) is a prevalent genetic disorder with global implications for severe cardiovascular diseases. Motivated by the growing recognition of the need for early diagnosis and treatment of FH to mitigate its severe consequences, alongside the gaps in understanding the economic implications and equity impacts of FH screening, this study aims to synthesize the economic evidence on the cost-effectiveness of FH screening and to analyze the impact of FH screening on health inequality.

**Methods:**

We conducted a systematic review on the economic evaluations of FH screening and extracted information from the included studies using a pre-determined form for evidence synthesis. We synthesized the cost-effectiveness components involving the calculation of synthesized incremental cost-effectiveness ratios (ICERs) and net health benefit (NHB) of different FH screening strategies. Additionally, we applied an aggregate distributional cost-effectiveness analysis (DCEA) to assess the impact of FH screening on health inequality.

**Results:**

Among the 19 studies included, over half utilized Markov models, and 84% concluded that FH screening was potentially cost-effective. Based on the synthesized evidence, cascade screening was likely to be cost-effective, with an ICER of $49,630 per quality-adjusted life year (QALY). The ICER for universal screening was $20,860 per QALY as per evidence synthesis. The aggregate DCEA for six eligible studies presented that the incremental equally distributed equivalent health (EDEH) exceeded the NHB. The difference between EDEH and NHB across the six studies were 325, 137, 556, 36, 50, and 31 QALYs, respectively, with an average positive difference of 189 QALYs.

**Conclusions:**

Our research offered valuable insights into the economic evaluations of FH screening strategies, highlighting significant heterogeneity in methods and outcomes across different contexts. Most studies indicated that FH screening is cost-effective and contributes to improving overall population health while potentially reducing health inequality. These findings offer implications that policies should promote the implementation of FH screening programs, particularly among younger population. Optimizing screening strategies based on economic evidence can help identify the most effective measures for improving health outcomes and maximizing cost-effectiveness.

**Supplementary Information:**

The online version contains supplementary material available at 10.1186/s41256-024-00382-x.

## Introduction

Familial hypercholesterolemia (FH) is an autosomal dominant inherited condition that accelerates the development of atherosclerotic cardiovascular disease and coronary artery disease, significantly increasing the risk of premature death [1]. Despite an estimated global prevalence of 1 in 200 individuals, most cases remain undiagnosed [2]. Early detection and timely pharmacological interventions can reduce the risk of myocardial infarction in FH patients by up to 76% and prevent early atherosclerosis, enabling a normal life expectancy [3]. Thus, conducting cost-effectiveness analyses of FH screening strategies is crucial to optimizing early diagnosis and treatment, which can significantly reduce the health and economic burden associated with FH [4].

Some national health authorities and professional medical organizations are increasingly emphasizing FH screening, endorsing actions through the formulation of guidelines and expert consensus. For instance, the National Institute for Health and Clinical Excellence (NICE) unveiled a United Kingdom (UK) guideline in 2008 for the identification and management of FH [5]. Similarly, the European Atherosclerosis Society's FH Studies Collaboration has emphasized the need for a global registry for FH, advocating for coordinated global initiatives [6]. The Australasia Network Consensus Group has also established new directives to guide clinicians in managing FH [7]. Furthermore, Northern Ireland, Scotland, and Wales have pioneered national FH services [8]. While the importance of FH screening is gaining recognition, there remains a significant gap concerning its economic implications, particularly the lack of comprehensive research on whether FH screening should be included in health insurance schemes.

Health inequality is a critical policy concern in many healthcare systems, necessitating an evaluation of the distribution of health costs and outcomes among diverse populations [9, 10]. Despite persistent calls for health technology assessment (HTA) agencies to incorporate equity evaluations into their decision-making processes, there remains a lack of research on the impact of FH screening on health inequality. The aggregate Distributional Cost-Effectiveness Analysis (DCEA) method incorporates equity considerations into traditional health economic evaluations, allowing for a comprehensive exploration of the impact of FH screening strategies on health inequality [11]. Considering these gaps, this study aims to synthesize the economic evidence on the cost-effectiveness of FH screening through a systematic review and to examine the impact of FH screening on population health inequality through an aggregate DCEA.

## Methods

### Study design

**Fig. 1:**
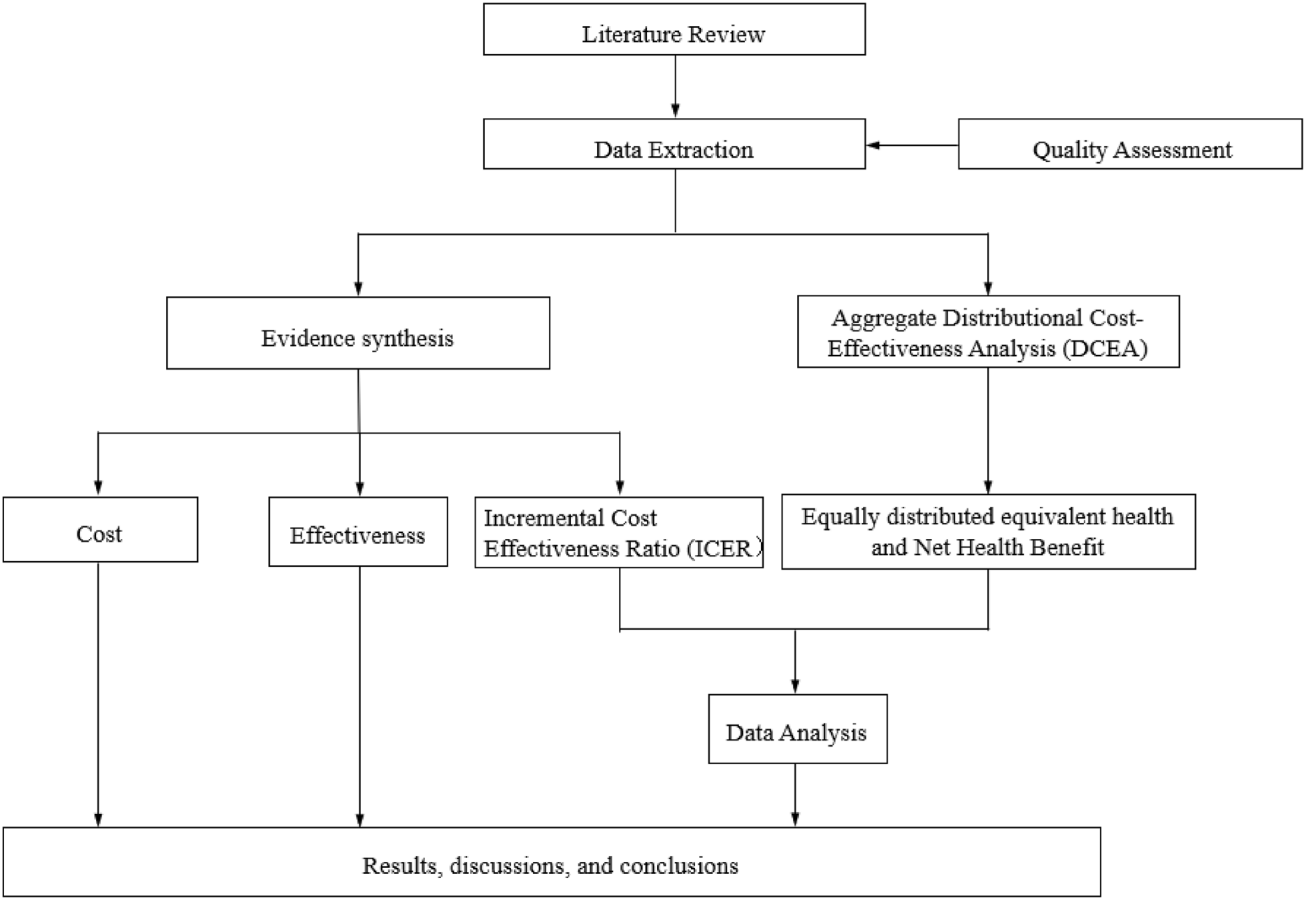
Study design

We conducted a systematic review to examine the cost-effectiveness evidence for FH screening strategies. As illustrated in Fig. 1, the study began with a thorough literature review followed by data extraction and quality assessment. Synthesized evidence from the included studies was used to evaluate both the cost and effectiveness of different FH screening approaches. In parallel, an aggregate distributional DCEA was performed to quantitatively assess the impact of FH screening on health inequality. This process involved calculating equally distributed equivalent health and net health benefits, leading to a comprehensive analysis of both the economic and distributional impacts.

### Systematic review

*Search strategy.* In this study, we employed a literature search to systematically review and analyze the economic evaluations of FH screening. We used key terms and corresponding MeSH terms such as "familial hypercholesterolemia," "cost-effectiveness analysis," "disease screening," and "health economics" to search multiple important databases, including PubMed, Web of Science, Embase, ScienceDirect, and the Health Technology Assessment Database. The search cutoff date was October 20, 2023, and the literature was limited to publications from 2000 onwards. Detailed search terms are provided in Table S1 of Supplementary Methods and Materials.

*Inclusion and exclusion criteria.* Predefined inclusion criteria were: (1) studies involving patients with FH, (2) studies focusing on FH screening, (3) studies conducting economic evaluations of screening methods, and (4) articles published in English. Exclusion criteria were: (1) studies involving patients with multiple diseases, (2) studies where the primary focus was not disease screening, (3) studies that did not report the process or results of economic evaluations, and (4) reviews, conference papers, and guidelines. Two authors (MW and JL) independently screened the literature for eligibility based on titles and abstracts in the first round and examined the full texts of potentially eligible articles in the second round to determine the final inclusion. Discrepancies were resolved through discussion with SJ and YG to achieve consensus.

*Data extraction.* We used a pre-determined form to extract information from the included studies. The extracted information included variables such as perspective, currency unit, screening strategies, screening targets, treatment drugs, economic evaluation methods, cost-effectiveness analysis results, and intricate details regarding the decision models employed (Tables 1 and 2).

**Table 1:** Characteristics and literature quality evaluation of FH screening studies included in the review

Country, References	Strategy type	Strategy number	Screening population	Definition of FH	Treatment	Economic evaluation method	Model	Time horizon	Cost unit	Discount rate	Perspective	QHES score	CHEERS 2022
UK,Kerr [24]	CS	2	FH patients and their relatives	Genetic testing	Statins,Ezetimibe	CEACUA	Markov	Lifetime	2014–15 UK pounds	3.5%	UK NHS perspective	87	22.5
UK, Crosland [25]	CS	9	Patients aged 40–70 years with FH and relatives	Genetic testing	Statins	CUA	Decision tree + Markov model	Lifetime	2015–16 UKpounds	3.5%	UK NHS perspective	91	22
UK, McKay [26]	US + RCT	8	Children 1–2 years old	Genetic testing; LDL-C testing;	Statins	CUA	Decision tree + Markov model	Lifetime	2017 UK pounds	3.5%	UK NHS perspective	86	21
UK,Marks [27]	US;CS;OS;	5	General population; Counseling patients; Inpatients with premature MI; FH patients and their relatives; People aged 16–54;	Genetic testing;LDL-C testing;	Statins	CEA	Decision tree + life-table analysis	Lifetime	pounds	costs:6% benefits:1%	UK NHS perspective	82	20
UK,Marks [28]	US;CS;	2	16-year-olds; Patients aged 16–54 years and their relatives	Genetic testing;LDL-C testing;	Statins	CEA	Decision tree + life-table analysis	10 years	pounds	Not Reported	UK NHS perspective	80	19
UK,Nherera [29]	CS	4	FH patients (50 years old) and relatives (30 years old)	LDL-C testing	Statins	CUA	Decision tree + Markov model	Lifetime	2010–11 UKpounds	3.5%	UK NHS perspective	89	22
Australia, Ademi [40]	CS	2	Ten years olds suspected of having FH	Genetic testing;LDL-C testing;	Statins	CEACUA	Markov	Lifetime	2019 Australian dollars	5%	Australian healthcare system perspective	91	23.5
Australia, Ademi [42]	CS	2	FH patients and their relatives	Genetic testing;LDL-C testing;	Statins	CEACUA	Decision tree + Markov	10 years	2013Australian dollars	5%	Australian health care perspective	88	20.5
Australia, Marquina [30]	US	2	Population 18–40 years in Australia	Genetic testing	Statins;Ezetimibe	CEACUA	Decision tree + Markov	Lifetime	2020Australian dollars	5%	Australian healthcare andsocietal perspective	93	24.5
U.S.Chen [37]	CS	3	Caucasian male adults	Genetic testing;LDL-C testing;	Statins	CUA	Decision tree + Markov	Lifetime	2013U.S. dollars	3%	U.S. societal perspective	88	21
U.S.Spencer [19]	US	2	Population-wide (20 and 35 years old)	Genetic testing	Statins	CEACUA	Decision tree + Markov	Lifetime	2021 U.S.dollars	3%	U.S. healthcare sector perspective	90	22.5
U.S.Jackson [33]	CS	2	FH + progenitor population, and the children of the progenitor population, and subsequent off-spring	Genetic testing	Statins,Ezetimibe, PCSK9	CEA	Simulated family trees;	30 years	2018U.S. dollars	3%	U.S. health care provider’sperspective	81	21.5
Argentina,Araujo [39]	US	2	Living in Argentina aged 6 years children	LDL-C testing	Statins	CEACUA	Decision tree	60 years	U.S.dollars	5%	Argentine public healthcare system perspective	84	21
Poland,Pelczarska [41]	US + CS;OS + CS;	7	People who got their first job; 6 years old children; 49/75 years of age after the first ACS/stroke;	Genetic testing;LDL-C testing;	Statins	CEACUA	Decision tree + Markov	Lifetime	zloty and euros	Costs:5% benefits:3.5%	Poland public payer perspective	89	21
Spanish,Lázaro [31]	CS	2	FH patients and their relatives	Genetic testing	Statins;Ezetimibe	CEACUA	Decision tree	10 years	2016 euros	3%	Spanish health system and social perspective	88	21
Spanish, Oliva [34]	CS	2	Under 60 years old FHand relatives	Genetic testing	Statins	CEA	Life-table analysis	Lifetime	2005euros	3%	Spanish health system perspective	89	21.5
Netherlands, Wonderling [35]	CS	2	FH patients and their relatives	Genetic testing	Statins	CEA	Life-table analysis	Lifetime	2001 U.S. dollars	4%	Netherlands health care perspective	85	20
Netherlands, Marang-van [36]	CS	10	FH patients over 16 years of age and their relatives	Genetic testing;LDL-C testing;	Statins	CEA	Life-tableanalysis	Lifetime	2002euros	Withoutdiscounting	Netherlands health care perspective	82	20.5
Netherlands, Ademi [32]	CS	2	10-year-olds FH	Genetic testing	Statins	CEACUA	Markov	Lifetime	2020euros	Costs:4%benefits:1.5%	Netherlands health care and societal perspectives	89	21.5

FH: Familial hypercholesterolemia; CS: Cascade screening; US: Universal screening; OS: Opportunistic screening; RCT: Reverse cascade testing; ACS: Acute coronary syndrome; MI: Myocardial infarction; CEA: Cost effectiveness analysis; CUA: Cost-utility analysis; LDL-C: Low-density lipoprotein cholesterol;

**Table 2:** Summary of economic results included in the review

References	Outcome measure	Cost (2023 US dollar)	Health benefit	ICERs (2023 US dollar)	WTP per QALY gained	Cost effectiveness
Kerr [24]	QALY CE	Net marginal cost per relative tested:4,702;	QALY per tested relative gain:0.48; Adverse events:46 MIs,50 cases of angina,8 strokes and 16 deaths;	9,818.19/QALY	£20,000/QALY£30,000/QALY	✓
Crosland [25]	QALY	Total costs:(1)11,444;(2)11,521;(3)11,595;(4)11,588;(5)11,792.87;(6)11,756;(7)11,866;(8)11,822;(9)11,536;	QALYs:(1)11.4079; (2)11.41755;(3)11.46357;(4)11.46325;(5)11.41999;(6)11.41991;(7)11.46601;(8)11.4657;(9)11.45383;	(2) vs (1)7,980.7/QALY(3) vs (4)22,502.73/QALY(4) vs (9)5,478.78/QALY(9) vs (1)1,996.88/QALY	£15,000 − £30,000/QALY	✓
McKay [26]	QALY;	Total costs(1)364,767;(2)905,645;(3)1,033,512;(4)1,085,284;(5)4,432,244;(6)4,498,466;(7)4,557,261;(8)4,623,484;	OALYs:(1)992.2;(2)1,009.1;(3)1,010.7;(4)1,027.5;(5)1,000.7;(6)1,022.2;(7)1,011.5;(8)1,033;	(2) vs (1)31,149.61/QALY (3) vs (1)35,304.4/QALY (4) vs (1)20,144.43/QALY (5) vs (1)458,090.37/QALY (6) vs (1)135,974.87/QALY (7) vs (1)212,476.88/QALY (8) vs (1)103,235.34/QALY(8) vs (4)644,978.34/QALY	£20,000/QALY £30,000/QALY	✓
Marks [27]	LYG	Cost per case detected (NA)	Gain in life years:7 years in men and 9.1 years in women aged 16–24 years	Clinical / Genetic(1)29,100.35/174,347.48/LYG(2)25,260.95/156,365.52/LYG (3)20,729.17/47,140.38/LYG (4)6,917.17/10,975.45/LYG (5)6,202.45/33,149.7/LYG	NA	✓
Marks [28]	CE	Total cost:(1)13,500,753;(2)101,486,932;	Averted deaths:(1)11.7deaths (male9.8, female1.9);(2)560 deaths (male377, female182)	(1)1,153,911.14/CE (2)181,224.63/CE	NA	✓
Nherera [29]	QALY	Total Cost(1)80,842;(2)92,344; (3)95,521;(4)99,382;Incremental cost:(2) vs (1)11,499; (4) vs (2)7,038;	QALYs:(1)10.89;(2)24.12;(3)24.28;(4)25.18;Incremental QALY:(2) vs (1)13.23;(4) vs (1)1.06;	(2) vs(1)868.71/QALY (4) vs (2)6,648.61/QALY	£20,000/QALY	✓
Ademi [40]	LYGQALYCE	Total costs(1)10,190,756;(2)10,674,822;net reduction cost: -852	QALY gained per person:(1) vs (2):1.07LYG per person (1)vs(2):0.97;Averted 24.2 acute non-fatal events;7.55 death;	ICER/QALY: Dominant ICER/LYG: Dominant	AUD$28,000/QALY	✓
Ademi [42]	LYG QALY CE	Incremental cost:84,620	LYG:(1)784.78;(2)759.83incremental LYG:24.95;QALY:(1)781.13;(2)752.06;incremental QALY:29.07;	(1) vs (2):3,391.93/LYG;2,910.99/QALY	AUD $6,000/QALY	✓
Marquina [30]	LYG QALY CE	Healthcare:Total costs:(1)2,391,377,517; (2)1,329,832,917;Incremental cost:1,061,544,600;Societal: (1)4,898,272,968 (2)6,692,327,539Incremental cost: -1,794,054,570;	Total years of life lived:(1)577,088;(2)543,600;incremental LYGs:33,488;Total QALYs:(1)503,500;(2)451,711;incremental QALY:51,790;Prevented 3,093 CHD event;	Healthcare perspective:(1) vs (2)31,698.75/LYG; 20,496.99/QALY;Societal perspective: ICER/LYG: Dominant ICER/QALY: Dominant	AUD$28,000/QALY and AUD$50,000/QALY	✓
Chen [37]	QALY	Total costs:(1)12,461;(2)19,640;(3)18,692;Incremental costs:(2) vs (1)7,179;(3) vs (1)6,230;	Total QALYs:(1)18.28;(2)18.77;(3)18.29;Incremental QALYs (2) vs (1)0.49;(3) vs (1)0.01;	(2) vs (1)14,651.74/QALY (3) vs (1)623,101.28/QALY(2) vs (3)1,975.46/QALY	$150,000/QALY	X
Spencer [19]	LYG QALY	20-year-olds: Total costs:(1) 2,551,478,334;(2)2,530,641,435;Incremental Cost:20,836,900;35-year-olds: Total Costs:(1)3,519,352,312;(2) 3,498,515,413; Incremental Cost: 20,836,899	20-year-olds:Incremental QALY:111;Incremental Life Years:69;35-year-olds:Incremental QALY:84;Incremental Life Years:54;	(1) vs (2)20 years old:188,573.94/QALY35 years old:243,791.72/QALY	$50,000, $100,000 and $150,000/QALY	X
Jackson [33]	LYG	FH Genetic Test:276 per relative	1st degree relatives:age < 40 average LYG: positive2st degree relatives:age < 15 average LYG: positive 3st degree relatives:Age = 5 average LYG: positive	positive	$50,000/QALY	✓
Araujo [39]	LYG QALY	NA	LYG each:8.14	1,762.29/LYG	NA	✓
Pelczarska [41]	LYG QALY	Incremental costs:(1)2,142,134;(2)3,393,626;(3)4,003,593.8;(4)2,305,455;(5)1,137,453;(6)11,197,272;(7)9,479,095;	LYG (total) (1)1,564;(2)1,650;(3)1,476;(4)915;(5)4,121;(6)1,049;(7)4,727;QALY (total) (1)1,450;(2)1,528;(3)1,371;(4)712;(5)3,774;(6)817;(7)4,329;	(1)1,369.48/LYG;1,477.19/QALY (2)2,056.14/LYG;2,221.55/QALY(3)2,711.38/LYG;2,920.39/QALY(4)2,519.68/LYG;3,236.48/QALY (5)276.33/LYG;301.34/QALY(6)10,669.22/LYG;1,3704.37/QALY(7)2,004.85/LYG;2,189.49/QALY	130,002PLN (29,800EUR)/QALY	✓
Lázaro [31]	CE QALY	Health care perspective: Direct costs total:(1)104,657,843;(2)65,065,947Incremental Cost:39,591,894;Societal perspective:Total costs:(1)147,451,737;(2)152,374,442;Incremental cost: -4,922,706;	Cardiac events(1)813;(2)1,661;event avoided:847;Coronary deaths:(1)196;(2)400;event avoided:203;QALYs:(1)62,175;(2)61,408;Incremental QALYs:767;	Healthcare perspective: 46,737/Cardiac event;194,621.77/Coronary deaths;51,649.33/QALY; Societal perspective:-5,810.72/Cardiac event;-24,198.85/Coronary deaths;-6,421.28/QALY;	€30,000/QALY	✓
Oliva [34]	LYG	Cost:(1)17,272;(2)8,349;Incremental cost:8,922;	Life Years:(1)56.7;(2)55.4;Incremental Life Years:1.34;	6,649.76/LYG	€10,000/QALY	✓
Wonderling [35]	LYG	Total incremental cost per new untreated case diagnosed:11,433;	LYG:0.90 (discounted);	13,452.45/LYG	NA	✓
Marang-van [36]	LYG	Current screening:(1)46,300,180;(2)31,235,074;(3)15,836,251;(4)28,593,017;(5)21,490,645;(6)9,649,819;Alternative screening(7)42,846,515;(8)21,170,891;(9)32,588,637;(10)14,644,281;	Current screening (LYG)(1)865; (2)610;(3)361;(4)519;(5)407;(6)204;Alternative screening(7)836;(8)507;(9)623;(10)337;	(1)53,509.08/LYG;(2)51,211.92/LYG (3)43,842.87/LYG;(4)55,056.49/LYG (5)52,795.28/LYG;(6)47,415.27 /LYG (7)51,278.68/LYG;(8)41,725.44/LYG (9)52,307.43/LYG;(10)43,409.79/LYG	€18,151/QALY	✓
Ademi [32]	LYG QALY	Health care perspectiveTotal health care costs per person:(1) 24,664,443;(2)8,613,984;Incremental:48,203;Societal perspectiveIncremental: − 193,386;	LYG per person:(1)38.78;(2):36.5;Incremental Life Years:2.28;QALY per person:(1)34.02;(2)31.48;Incremental QALYs:2.53;	Healthcare perspective:12,378.51/QALY; Societal perspective: DominantROI:11.24;	€20,000/QALY	✓

LYG: Life years gained; QALY: Quality-adjusted life years; CE: Events averted; ROI: Return on investment; WTP: Willingness to pay; ICERs: Incremental cost-effectiveness ratios

*Quality assessment.* To ensure the inclusion of high-quality economic evaluations in our review, we employed two established assessment tools: the Quality of Health Economic Studies (QHES) for assessing the quality of analysis [12, 13], and the Consolidated Health Economic Evaluation Reporting Standards (CHEERS) for assessing the quality of reporting [14]. The QHES tool assigns scores ranging from 0 to 100. We categorized the included studies as high quality (75–100), moderate quality (50–74), low quality (25–49), and very low quality (0–49). The CHEERS checklist consists of 28 checking items. Each item was scored as follows: 1 point for being fully satisfied, 0.5 points for partially satisfied, and 0 for unsatisfied, with a maximum possible score of 28 points.

### Evidence synthesis

Following the systematic review, we synthesized the evidence from studies that evaluated the same screening strategies (cascade or universal screening), utilized the same or similar outcome measures (adverse events averted, Life Years Gained, or Quality-Adjusted Life Years), and applied the same perspective (healthcare system or societal perspective). These criteria were applied to ensure that the evidence for synthesis was consistent.

*Cost synthesis.* To address the heterogeneity in currency usage and publication years across different articles, all costs were standardized to 2023 U.S. dollars using a web-based tool for cost conversion that applies purchasing power parities [15, 16]. For costs that did not report confidence intervals, we assigned an interval of ± 50% of the reported value. The types of costs considered in the included articles are detailed in Table S2 in the Supplements.

*Effectiveness synthesis.* We synthesized health outcomes across four categories: deaths averted, adverse events averted, Life Years Gained (LYGs), and Quality-Adjusted Life Years (QALYs). For outcomes that did not report confidence intervals, we assigned an interval of ± 50% of the reported value to account for uncertainty in the outcome parameters. In one study, results were provided for two cohorts (20-year-olds and 35-year-olds); we combined these cohorts to derive an average outcome for the two age groups [17].

*Cost-effectiveness analysis.* With the synthesized costs and health outcomes, we calculated the cost-effectiveness of each screening strategy compared with no screening or *status quo*. We used the Incremental Cost Effectiveness Ratio (ICER) as the indicator of being cost-effective, which was determined by dividing the incremental costs by the incremental health outcomes between the examined screening strategy relative to its comparator. Based on the confidence intervals for costs and health outcomes, we calculated the confidence intervals for ICERs using the Delta method to account for uncertainty (Table 3) [18].

**Table 3:** Synthesis of cost-effectiveness analysis results and COMER outcomes

Category	References	Incremental costs(2023 US dollar)	Incremental effects	Total ICER(2023 US dollar)	NHB(2023 US dollar)	NHB < 0(%)	Weight (%)
CS(QALY)	Kerr [24]	4,702.79	0.48	49,630(37,223 to 62,038)	19,648	NHB > 0	0.4734
	Ademi [40]	− 852.31	1.07		23,369	NHB > 0	0.3346
	Ademi [42]	84,620.28	29.07		57,823	NHB > 0	0.0547
	Lázaro [31]	39,591,894.47	767		547,516	NHB > 0	0.0006
	Ademi [32]	31,369.18	2.53		36,563	NHB > 0	0.1367
		$\Sigma \omega *c$=34,991	$\Sigma \omega *e$=2.99		TNHB = 25,614(19,210 to 32,017)	TNHB > 0	$\Sigma w$=1
CS(LYG)	Ademi [40]	− 852.31	0.97	4,451(3,338 to 5,564)	21,264	NHB > 0	0.225
	Ademi [42]	84,620.28	24.95		37,634	NHB > 0	0.0718
	Oliva [34]	8,922.69	1.34		17,108	NHB > 0	0.3477
	Wonderling [35]	11,433.06	0.9		20,539	NHB > 0	0.2412
	Ademi [32]	31,369.18	2.28		29,851	NHB > 0	0.1142
		$\Sigma \omega *c$=15,331	$\Sigma \omega *e$=2.95		TNHB = 21,801(16,351 to 27,251)	TNHB > 0	$\Sigma w$=1
CS (adverse events averted)	Kerr [24]	4,702.79	104	406,03(30,452 to 50,753)	5,271,321	NHB > 0	0.0092
	Ademi [40]	− 852.31	24.2		510,117	NHB > 0	0.9795
	Lázaro [31]	39,591,894.47	847		4,734,156	NHB > 0	0.0114
		$\Sigma \omega *c$=449,450	$\Sigma \omega *e$=34.29		TNHB = 601,825(451,368 to 752,281)	TNHB > 0	$\Sigma w$=1
CS (deaths averted)	Kerr [24]	4,702.79	16	179,369(134,526 to 224,211)	806,993	NHB > 0	0.0377
	Marks [28]	101,486,932.5	560		− 64,766,053	NHB < 0	0.000005
	Ademi [40]	− 852.31	7.55		159,735	NHB > 0	0.9633
	Lázaro [31]	39,591,894.47	203		− 28,968,295	NHB < 0	0.00003
		$\Sigma \omega *c$=1,110	$\Sigma \omega *e$=7.88		TNHB = 182,905(137,178 to 228,631)	TNHB > 0	$\Sigma w$=1
US(QALY)	Marquina [30]	1,061,544,599.77	51,790	20,860(15,645 to 26,075)	854,219,290	NHB > 0	0.00004
	Spencer [19]	20,836,899.42	97.5		− 5,599,989	NHB < 0	0.99996
		Σω*c = 20,881,624	$\Sigma \omega *e$=99.72		TNHB = − 5,563,039(− 6,953,798 to − 4,172,279)	TNHB < 0	$\Sigma w$=1
US (deaths averted)	Marks [28]	13,500,753.09	11.7	832,917(624,687 to 1,041,146)	− 12,733,549	NHB < 0	0.99998
	Marquina [30]	1,061,544,599.77	1,279		− 1,014,233,111	NHB < 0	0.00002
		$\Sigma \omega *c$=13,665,924	$\Sigma \omega *e$=11.9		TNHB = − 12,891,385(− 16,114,231 to − 9,668,539)	TNHB < 0	$\Sigma w$=1
US(LYG)	Marquina [30]	1,061,544,599.77	33,488	32,262(24,197 to 40,328)	177,210,008	NHB > 0	0.004
	Spencer [19]	20,836,899.42	61.5		− 11,225,925	NHB < 0	0.996
		$\Sigma \omega *c$=24,996,554	$\Sigma \omega *e$=195.1		TNHB = − 10,472,757(− 13,090,946 to − 7,854,568)	TNHB < 0	$\Sigma w$=1

CS: Cascade screening; US: Universal screening; ω: weigh; Σω*c: The weighted sum of costs; NHB: Net health benefit; TNHB: Total health benefit; LYG: Life years gained; QALY: Quality-adjusted life years; CE: Events averted

*Total net health benefit.* To synthesize data from different age groups across different studies, we employed the Comparative Efficiency Research (COMER) approach (Appendix 1 of Supplementary Methods and Materials) to generate weights for each group within each study [19]. These weights were applied to calculate the weighted incremental costs and incremental health outcomes for each age group across the studies (Table 3).

### Aggregate distributional cost-effectiveness analysis

We conducted an aggregate DCEA based on the cost-effectiveness analysis results to evaluate the distribution of health outcomes and costs following FH screening across diverse population groups. This analysis was aimed at estimating the impact of FH screening on health inequality. The process was carried out following several key steps as described below.

*Step 1: initial health distribution.* The first step involved estimating the distribution of health outcomes prior to the implementation of FH screening across different population groups. The baseline distribution of health was from a previous study, which offered detailed data on the distribution of quality-adjusted life expectancy (QALE) at birth across different socioeconomic groups [20]. The socioeconomic status was measured by the Index of Multiple Deprivation (IMD), which categorises the population into five groups: IMD1 to IMD5. IMD1 represents the most deprived areas, while IMD5 represents the most affluent areas [14]. According to their results, the baseline QALE was 63.21 years for IMD1 and 75.00 years for IMD5 (Table S3 in Supplements).

*Step 2: initial health opportunity cost distribution*. In this step, we applied the health opportunity cost distribution across population groups. The health opportunity cost represents the health benefits that are foregone when resources are reallocated within the healthcare system, i.e. the health benefits derived from a treatment that the poorest population group would forego if decision-makers decide to fund a different treatment. The distribution of health opportunity costs represents how these forgone health benefits are spread across different population subgroups, such as the IMD groups. For example, if a 20% opportunity cost is attributed to the IMD1 group (most deprived people), it means that if decision-makers choose to fund a different treatment, 20% of the total health benefits forgone will be born by this group. This distributional information helps decision-makers identify which groups are most adversely affected by funding decisions and assess the equity implications of different healthcare interventions. In our analysis, we used data from a previous study that quantified the distribution of health opportunity costs among IMD groups [21]. The study found that 26% of the health opportunity costs were borne by IMD1, while 14% were borne by IMD5 (Table S3 in Supplements).

*Step 3: net health benefit.* In the third step, we calculated the net health benefit (NHB) derived from the intervention under consideration—in this case, FH screening. The NHB provides a measure of the health benefits gained from the intervention after accounting for the health benefits potentially lost due to the reallocation of healthcare resources (i.e., opportunity cost). Our NHB calculation procedure was: (1) we calculated the total health benefits within each IMD group. This was determined by multiplying the incremental QALYs gained from the intervention by the number of patients in that group. (2) we estimated the opportunity cost within each group, which represents the potential monetary benefits that could be forgone if resources are diverted to FH screening. The opportunity cost for each group was calculated by multiplying the incremental cost per patient by the total number of patients in the group and then multiplying this result by the proportion of opportunity cost specific to that group (as derived in Step 2). (3) the potential health benefits forgone due to the opportunity cost were then calculated by dividing the within-group opportunity cost by the opportunity cost threshold. This threshold reflects the value of health benefits that are sacrificed when resources are diverted from their existing uses to fund a new intervention. (4) the NHB for each group was determined by subtracting the potential health benefits forgone due to the opportunity cost from the total health benefits gained from FH screening. The resulting NHB represents the net health gains from the intervention after considering the cost of health benefits lost elsewhere due to resource reallocation. The calculation formula used is as follows:

1 $$\begin{array}{cc}NH{B}_{j}& =(\Delta QALY\cdot {n}_{j})-(\frac{N\cdot \Delta cost\cdot {d}_{j}}{K})\end{array}$$

where ${NHB}_{j}$ is the net health benefit for the ${j}^{th}$ group; $\Delta QALY$ denotes the incremental health gains derived from FH screening; ${n}_{j}$ is the number of patients screened in the ${j}^{th}$ group; $N$ is the total number of patients screened;${d}_{j}$ is the opportunity cost proportion for the ${j}^{th}$ group; $\Delta cost$ refers to the incremental cost of FH screening per patient relative to comparator; $K$ is the opportunity cost threshold per patient, reflecting the value of health benefits forgone when resources are allocated to this intervention. The opportunity cost threshold is typically set equal to the societal willingness-to-pay (WTP) threshold, which is the benchmark used to determine whether the health gains from a new intervention justify the health losses incurred elsewhere. For instance, NICE sets the threshold at £20,000-£30,000 per QALY. In our study, we used the societal WTP threshold specified in each study eligible for aggregate DCEA calculation.

*Step 4: post-intervention QALE*. In this step, we integrated the NHB derived from FH screening (as calculated in Step 3) into the initial health distribution for each group to produce the post-intervention health distribution. The initial health distribution, expressed as QALE, was updated by adding the NHB, resulting in the post-intervention QALE. This updated QALE reflected the net impact of FH screening. Meanwhile, the NHB represented the incremental QALE between the pre- and post-intervention scenarios.

*Step 5: pre- and post-intervention equally distributed equivalent health.* In this step, we calculated the Equally Distributed Equivalent Health (EDEH) both before and after intervention. EDEH represents the mean level of health per person that, if equally distributed across the population, would give the same level of societal welfare as the current unequal distribution. To estimate EDEH, we used the Atkinson inequality index, which measured the level of inequality in a health distribution. The Atkinson index is calculated using the following formula:

2 $$A\left(\epsilon \right)=1-{\left(\frac{1}{N}\sum_{i=1}^{N}{\left(\frac{{h}_{i}}{h}\right)}^{1-\epsilon }\right)}^{\frac{1}{1-\varepsilon }}$$

where $A(\varepsilon )$ is the Atkinson Inequality Index; $N$ represents the number of population groups; ${h}_{i}$ is the QALE within the ${i}^{th}$ group; $h$ is the average QALE of the entire population;$\varepsilon$ is the inequality aversion parameter, which was set at 10.95 based on a previous empirical measurement in the UK [23]. Using the QALE values by group and the average QALE for the pre-intervention scenario, we calculated the pre-intervention Atkinson index of inequality. Similarly, by using the QALE values by group and the average QALE post-intervention (adjusted by the NHB derived in Step 3), we calculated the post-intervention Atkinson index of inequality. We used the two Atkinson indexes to generate EDEH both before and after intervention. The $EDEH$ was calculated using the following formula:

3 $$EDEH=N\cdot \left(1-A\left(\varepsilon \right)\right)\cdot h$$

where $h$ is the average QALE of the population, and $N$ is the total number of patients screened. The difference between pre- and the post-intervention EDEH provided the incremental EDEH.

*Step 6: population equity impact.* In the final step, we assessed the population equity impact of FH screening by comparing the incremental QALE (equivalent to the NHB) with the incremental EDEH. The population equity impact was calculated by subtracting the incremental QALE from the incremental EDEH. This calculation allowed us to derive the net equity impact by FH screening. A positive value indicated a reduction in health inequality, as the distribution of health outcomes became more equal. Conversely, a negative value indicated an increase in health inequality, meaning the distribution of health outcomes became more unequal [14].

## Results

### Characteristics of included studies

**Fig. 2:**
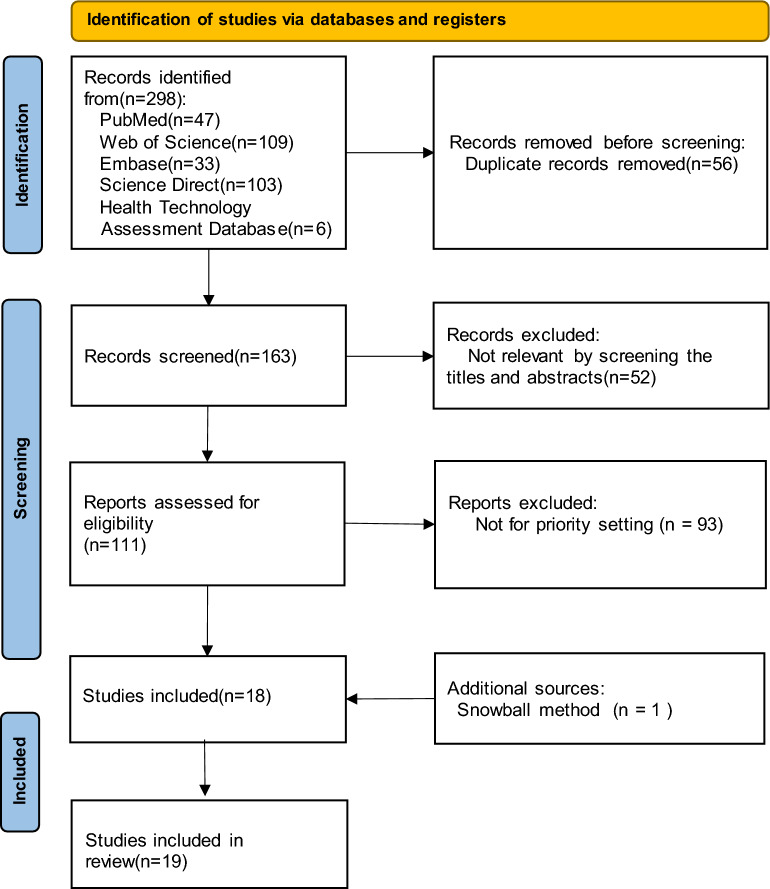
Literature screening and selection process

We identified a total of 19 articles for the review process (Fig. 2). A total of 79% of studies applied a healthcare system perspective, considering only direct medical costs, while 16% of studies applied both the healthcare system and societal perspectives (Table 1). Modelling approaches included Markov models (the most common, comprising over half the studies), decision trees, life-table analysis, and simulated family trees (Appendix 2).

### Quality assessment outcomes

The quality assessment of the included studies yielded an average QHES score of 87, with a range of 80–93, indicating that all selected studies met the high-quality standard. Furthermore, the CHEERS 2022 assessment on the reporting quality yielded an average score of 21 with scores ranging from 19 to 24.5 (Table 1). Details of the quality assessment are provided in Tables S4 and S5 in Supplements.

### Cost-effectiveness of FH screening

Most (17 out of 19) FH screening studies reported ICER values below their respective country's willingness-to-pay thresholds, indicating cost-effectiveness. However, two U.S. studies found FH genomic screening not cost-effective at current thresholds [17, 22]. To enhance model robustness, 95% of studies conducted sensitivity analyses, with 53% using probabilistic sensitivity analysis and presenting cost-effectiveness acceptability curves (CEACs). Cost-effectiveness probabilities varied significantly with willingness-to-pay thresholds. For U.S. population-wide genomic screening of 20-year-olds, FH screening probabilities were 1%, 38%, and 81% at QALY thresholds of $50,000, $100,000, and $150,000, respectively. For 35-year-olds, these probabilities were 0%, 14%, and 57% [17].

### Cost-effectiveness of screening during childhood

FH screening of children showed favourable cost-effectiveness in several countries [23]. In the UK, McKay et al. found that universal screening of 1–2-year-olds followed by reverse cascade testing was cost-effective [24]. In Argentina, a probabilistic model assessed the expected cost-effectiveness of universal FH screening for 6-year-old children, revealing it as a highly cost-effective health technology [25]. Similar studies were conducted in Australia and the Netherlands. Ademi et al. estimated the cost-effectiveness of cascade screening for 10-year-old children from the perspectives of the Australian public healthcare system and Dutch healthcare and society, respectively. [26, 27] The results consistently showed that cascade screening for 10-year-old children was cost-effective compared to current healthcare in both Australia and the Netherlands.

### Cost-effectiveness by age

The cost-effectiveness of FH screening varied by age. In the United States, comprehensive genomic FH screening found improved cost-effectiveness for screening younger patient cohorts compared to older ones [17]. An Australian study conducted a cost-effectiveness assessment of genomic screening for young individuals with FH [28]. Subgroup analysis revealed that narrowing the screening age range from 18–40 years to 18–25 years resulted in an increased cost per QALY gained. Another Australian study showed that screening 10-year-olds for FH and starting statin therapy immediately was cost-saving compared to screening 18-year-olds [27].

### Cost-effectiveness of cascade screening

The cost-effectiveness of cascade screening was evident in several countries. Some countries integrated cascade screening with different case identification methods to determine the most cost-effective screening strategy. These methods included searching electronic health records, utilizing various clinical assessment standards [29], screening identified cases separately based on genetic testing and cholesterol testing [30], and combining genetic testing and cholesterol testing but distinguishing the order [24]. Results showed that combining these diverse case identification methods was more cost-effective than using cascade testing alone [29].

### Cost-effectiveness of screening strategy combinations

Recognizing the complementarity of these strategies, some countries explored combined approaches for more comprehensive FH screening. In Poland, researchers combined universal screening followed by cascade screening for different populations or opportunistic screening followed by cascade screening for clinically or genetically diagnosed high-risk populations. Evaluations of seven strategies showed that screening patients with acute coronary syndrome under 55–65 years using clinical criteria emerged as the most cost-effective strategy. From the perspective of public payers, a combination of multiple strategies might be the most acceptable solution for implementing FH screening [31].

### Evidence synthesis

FH screening was deemed potentially cost-effective in 84% of studies (Table 2). After excluding studies lacking specific cost or outcome values and those with incomparable screening strategies, the final synthesis included eight studies on cascade screening (Table S6 in Supplements) and three studies on universal screening (Table S7 in Supplements). These studies considered outcome measures such as QALYs, LYGs, adverse events averted, and deaths averted, resulting in the synthesis of seven distinct groups.

*Cost-effectiveness of cascade screening.* Synthesis of study results by outcome measure revealed varying cost-effectiveness (Table 3). For QALYs, the synthesized results indicated a total incremental cost of $39,711,734, a total incremental health gain of 800 QALYs, and an ICER of $49,630 per QALY. For LYGs, the total incremental cost was $135,493 with a gain of 30.44 LYGs and an ICER of $4,451 per LYG. For adverse events averted, the incremental cost was $39,595,745 with 975.2 events averted and an ICER of $40,603 per event averted. For deaths averted, the ICER was $179,369 per death averted.

*Cost-effectiveness of universal screening.* For studies using LYGs and QALYs as the outcome measures [17, 28], the synthesized results showed a total incremental cost of $1,082,381,499, with a total incremental LYG of 33,550 (ICER of $32,262 per LYG) and 51,878 QALYs (ICER of $20,860 per QALY). For studies using deaths averted as the outcome measure [28, 32], the total incremental cost was $1,075,045,353, total deaths averted was 1290.7, and the overall ICER was $832,917 per death averted.

*Total net health benefit.* Analysis of COMER results revealed contrasting total net health benefits across the seven categories (Table 3). Cascade screening groups consistently showed positive NHB: $25,614 for QALYs, $21,801 for LYGs, $601,825 for adverse events averted, and $182,905 for deaths averted. Conversely, universal screening groups displayed negative NHB: − $5,563,039 for QALYs, − $10,472,757 for LYGs, and − $12,891,385 for deaths averted. These findings, summarized in Table 3, suggest that cascade screening may offer more favourable health economic outcomes compared to universal screening in FH detection.

#### Aggregate distributional cost-effectiveness analysis

This study used a mean QALY of 69.72 per individual as the baseline EDEH, given an Atkinson inequality aversion parameter of 10.95. The aggregate DCEA results from six studies showed positive differences between the incremental EDEH and the NHB, indicating that the FH screening strategies in the six studies could reduce health (Table 4).

**Table 4:** The impact of FH screening strategies on health inequality

References	Δ cost	ΔQALY	WTP	Population number	ΔNHB	ΔEDEH	ΔEDEH −ΔNHB	Value
Kerr [24]	2,781	0.48	30,000	6,393	2,477	2,802	325	positive
Crosland [25]	45.772	0.00965	30,000	2,354	19	156	137	positive
McKay [26]	335,088	16.9	20,000	10,000	1,456	2,012	556	positive
Ademi [40]	− 1,134	1.07	28,000	1,000	1,111	1,147	36	positive
Chen [37]	5,989	0.49	150,000	1,000	450	500	50	positive
Ademi [32]	23,365	2.53	20,000	1,000	1,362	1,393	31	positive

Δ cost: Incremental cost; ΔQALY: Incremental quality adjusted life years; WTP: Willingness to pay; ΔNHB: Incremental Net Health Benefit; ΔEDEH: Incremental equally distributed equivalent health

Studies conducted in the UK demonstrated reductions in health inequality across various FH screening strategies. Cascade screening of FH had an incremental NHB of 2,477 QALYs and an incremental EDEH of 2,802 QALYs [33]. Another study on cascade screening showed a difference of 137 QALYs between incremental EDEH and incremental NHB [29]. Universal screening plus reverse cascade screening showed a difference of 556 QALYs [24].

Australia's FH cascade screening resulted in an incremental EDEH of 1,147 QALYs and an incremental NHB of 1,111 QALYs, indicating a reduction in health inequality [27]. In the US, the incremental EDEH was 450 QALYs, exceeding the NHB [34]. The Netherlands also showed a positive difference between incremental EDEH and NHB when using an opportunity cost threshold of €20,000 per QALY gained [26].

## Discussion

Although there are some reviews on the economic evaluation of FH screening, there is still a lack of comprehensive evidence synthesis and an investigation of its impact on health inequality. [22, 35] This study represented the first comprehensive synthesis of evidence on the economic evaluation of FH screening and explored the impact of implementing FH screening on reducing health inequality, filling a gap in the existing literature.

This study found significant heterogeneity among the included studies and highlighted the importance of considering a variety of factors in the economic evaluation of FH screening. First, the perspective of analysis was crucial [36]. Although most studies tended to analyze from the payer’s perspective, this often neglected a comprehensive assessment of productivity losses [37]. Specifically, we should evaluate the return on investment of FH screening from a broader socio-economic perspective [18]. Second, the choice of decision analysis model had a decisive impact on the study outcomes [36]. We noted that in these studies, the Markov model was widely used for its flexibility and was often combined with decision trees and other methods to more comprehensively capture the complexities related to FH screening [38, 39]. Third, choosing the tracking time frame flexibly based on specific research needs was extremely important for enhancing the practicality of the model [40]. Although many studies applied a lifetime horizon, considering different time spans such as 10 years, 30 years, or 60 years allowed the model to better adapt to different policy needs [32].

The demonstrated cost-effectiveness of cascade screening in an increasing number of countries highlights its importance [41]. However, the exploration of cascading through multiple generations remained an important avenue for investigation. A study in the US, simulating approximately 6 million individuals using the Simulation of Family Tree, revealed that beyond first and second-degree relatives, cascade screening was not cost-effective [42]. While many countries conducted cascade screening and demonstrated its cost-effectiveness, only a study in the UK applied reverse cascade screening, proving its economic effectiveness after universal screening for children. This underscored the importance of future discussions on the strategic integration of reverse cascade screening for FH in children [24, 36].

The crucial importance of determining the cost-effectiveness of health technology, particularly in the context of publicly funded healthcare insurance systems, cannot be overstated [50]. However, we observed that economic evaluations of FH screening were predominantly concentrated in developed countries, while such research was comparatively scarce in developing countries. As awareness of FH increased, more developing countries recognized the significance of FH screening and management in enhancing public health [43, 44]. Initiatives to assess the healthcare system's capability to manage FH patients were initiated in several developing countries, including Pakistan, India, and Malaysia [45–47]. Although the initial efforts in these countries had not yet encompassed a comprehensive economic evaluation of FH screening, our thorough synthesis of evidence offered informational support and served as a learning experience for these countries to advance their FH screening evaluations [48]. This is especially crucial in areas marked by limited resources and poverty, as it promises not only to improve the population health but also has the potential to reduce health inequality. Notably, our findings underscored the cost-effectiveness of FH screening in Argentina, a developing country, providing a promising outlook for other developing countries contemplating the implementation of FH screening programs [25].

Statins could reduce LDL-C levels by inhibiting cholesterol synthesis enzymes, thereby preventing FH effectively [49]. However, for FH patients requiring high-dose statin treatment yet intolerant to its side effects, PCSK9 inhibitors may emerge as a crucial alternative [50]. Although PCSK9 inhibitors demonstrated remarkable effectiveness in lowering LDL-C levels, their cost-effectiveness in patients with heterozygous FH did not meet the generally accepted incremental cost-effectiveness threshold [51]. The potential cost-effectiveness of screening plus PCSK9 treatment approaches remained unclear. It is imperative to consider them in the broader context of screening and treatment strategies in future economic evaluations.

Precision public health, aiming to provide the right intervention to the right population at the right time, was a continually evolving field [52]. The cost-effectiveness of genetic testing and cholesterol testing in FH screening economic evaluations varied between countries, influencing economic outcomes [51]. In a study conducted in the UK, all DNA-based methods were shown to be cost-effective compared to cholesterol-only methods [30]. However, in some US studies, the cost-effectiveness of genetic testing was challenged by a variety of factors such as the high costs associated with testing and a lack of data related to genomic findings [17, 34]. This underscored the necessity of careful consideration of complex factors involved in the application of genetics and genomics, such as the testing cost and its declining speed, secondary genomic findings, future related and unrelated medical costs, and the preferences of stakeholders [53–56].

This study has several limitations. First, the analysis of the impact of FH screening on health inequality relied on empirical evidence from the UK, such as the distribution of initial health and the opportunity cost proportions across population groups. Due to the lack of evidence on these parameters in other countries, our conclusions are based on UK-specific parameters, which may be different in other contexts, potentially leading to different outcomes of aggregate DCEA. Second, our evidence synthesis may be affected by biases existing in the included articles, as we were unable to recalibrate the original data to check for biases. Although we attempted to include only the high-quality articles in our analysis by using a double-quality evaluation method, this approach cannot fully verify the accuracy of the original data and conclusions. Third, our study included only one economic evaluation from a developing country, due to the limited evidence from other developing countries. This could lead to biased conclusions when attempting to generalize the findings that are mostly based on developed countries to the context of developing countries.

## Conclusions

Our research provided insights into the economic evaluation of FH screening strategies, revealing significant heterogeneity in the methods and outcomes across different contexts. Most studies demonstrated the cost-effectiveness of conducting FH screening. Moreover, FH screening not only contributed to the improvement of overall population health but also had the potential to reduce health inequality. This study provides important policy implications for the implementation of FH screening. First, policies should promote the early screening of FH, particularly targeting younger populations, to facilitate timely diagnosis and management of FH condition, thereby reducing future health burdens. Additionally, global collaboration is essential in developing tailored economic evaluations of FH screening that account for different national contexts and policy environments. By optimizing screening strategies based on economic evidence, policymakers can identify the most effective measures for improving health outcomes while ensuring cost-effectiveness.

## Supplementary Information

Additional file 1.
