# Supplementary material for: Synthesized economic evidence on the cost-effectiveness of screening familial hypercholesterolemia
Source: Glob Health Res Policy. 2024 Sep 26;9:38. doi: 10.1186/s41256-024-00382-x (PMC11425997; doi:10.1186/s41256-024-00382-x)
Supplement: Supplementary file 1 — Additional file 1. [file 41256_2024_382_MOESM1_ESM.docx]

**Supplementary Methods and Materials**

# Appendix 1

Comparative efficiency research (COMER)

In this appendix, we list the main component of comparative efficiency research (COMER) approach.^1^ In cost-effectiveness analysis, we calculate the incremental cost-effectiveness ratio (ICER), which is derived by:

$$ICER=\frac{c_{1}-c_{2}}{e_{1}-e_{2}}$$

(1)

where $c$is cost; $e$ denotes effectiveness; $1$and $2$ correspond to the intervention of interest and the baseline intervention as the comparator, respectively. In our context, they represent the FH screening strategy under investigation and the *status quo* strategy, respectively.

The net health benefit of a study is calculated by

$$NHB_{s}=k*\left( e_{s1}-e_{s2} \right)-\left( c_{s1}-c_{s2} \right)$$

(2)

The weight of a study is calculated by:

$$\omega_{s} = \frac{\frac{1}{var\left( NHB_{s} \right)}}{\sum_{i=1}^{S} \left( \frac{1}{var\left( NHB_{s} \right)} \right)}$$

$$=\frac{\frac{1}{\left[ k^{2}*Var\left( e_{s1}-e_{s2} \right)+Var\left( c_{s1}-c_{s2} \right)-2*k*Covariance\left( e_{s1}-e_{s2},c_{s1}-c_{s2} \right) \right]}}{\sum_{i=1}^{S} \left( \frac{1}{var\left( NHB_{s} \right)} \right)}$$

(3)

The total net health benefit (TNHB) is calculated by:

$$TNHB = \sum\omega_{s}*NHB_{s}$$

$$= \sum\omega_{s}*\left( k*\left( e_{s1}-e_{s2} \right)-\left( c_{s1}-c_{s2} \right) \right)$$

$$= k*\sum\omega_{s}*\left( e_{s1}-e_{s2} \right)-\sum\omega_{s}*\left( c_{s1}-c_{s2} \right)$$

(4)

where $s$ denotes study ID; 1 and 2 refer to the intervention of interest and the baseline intervention as the comparator; $c$is cost; $e$ denotes effectiveness; $k$ is the willingness to pay; $w$ is the weight of the study.

# Appendix 2

Supplementary results of systematic review

National and Analytical Perspective

This study conducted a comprehensive analysis of 19 literature pieces, encompassing diverse geographical focuses: 32% in the UK, 16% in Australia, 16% in the US, 5% in Argentina, 5% in Poland, 11% in Spain, and 16% in the Netherlands. Geographically, 63% concentrated in Europe, 16% in North America, 16% in Oceania, and 5% in South America, with no studies from Asia and Africa.

These countries approached economic evaluations from different perspectives. Specifically, 79% of the studies solely analyzed from a healthcare perspective, considering direct medical costs, including screening and treatment expenses. In contrast, 5% of the studies conducted analyses from a societal perspective, while an additional 16% simultaneously considered both healthcare and societal perspectives. These studies not only factored in medical costs but also took into account indirect costs arising from productivity loss.

Cost unit and discount

The currency units used are US dollar, Euro, Australian dollar and British pound. 32% reported pounds, 26% euros, 16% Australian dollars and 26% US dollars. 89% discount cost and benefits to reflect loss of value in future outcomes, and only 11% do not discount. In the discounted papers, 82% of the cost and benefits adopted the same discount rate, and 18% adopted different discount values respectively. The cost discount rate ranges from 3%-6%, and the benefits discount rate ranges from 1%-5%.

Economic Evaluation Methods, Outcome Measures, and Sensitivity Analysis

In the study, 32% of the literature used cost-effectiveness analysis (CEA) as measured by the number of adverse events avoided and years of life gained (LYG), while 21% used cost-utility analysis (CUA) as measured by quality-adjusted life years (QALY) for health outcomes. Additionally, 47% combined CEA and CUA for economic evaluation. 68% reported QALY, 63% reported LYG, and 32% reported avoided adverse events.21% reported QALY, LYG, and CE simultaneously. To ensure model robustness, 95% of studies conducted sensitivity analyses, with 42% using one-way sensitivity analyses and 53% utilizing both one-way and Probabilistic Sensitivity Analysis.

Decision Analysis Models and Time horizon

Various decision analysis modeling methods were employed, including Markov models, Decision trees, Life-table analysis, Simulated family trees, and combinations such as Decision tree + Markov model and Decision tree + Life-table analysis. Specifically, 16% used Markov models alone, 11% used Decision trees alone, 16% used Life-table analysis alone.
42% employed a combination of Decision tree + Markov model, and 11% combined Decision tree + Life-table analysis.58% of studies utilizing Markov models reported Health States, with health state numbers ranging from 3-14.

Regarding the timeframe, 74% of studies focused on a lifetime, while some considered different tracking durations, including 10, 30, and 60 years, with 16% analyzing a 10-year span.

FH Detection Methods, Sensitivity and Specificity, Treatment

Different detection methods were employed, with 47% using gene testing, 5% using LDL-C (low-density lipoprotein cholesterol) testing, and 47% using both gene and LDL-C testing, demonstrating effectiveness.

During testing, 53% of studies reported gene testing sensitivity (ranging from 78.5% to 100%), and 47% reported specificity (ranging from 99.7% to 100%). In articles reporting both sensitivity and specificity, 32% assumed both to be 100%.

Post-screening treatment for FH patients commonly involved Statins (84%), followed by combinations like Statins and Ezetimibe (11%), and Statins, Ezetimibe, and PCSK9 (5%). Regarding adherence to lipid-lowering therapy, 21% of studies assumed 100% adherence.

# Supplementary Tables

Table S1. Search terms and results in five databases.

| Pubmed | | |
| --- | --- | --- |
|  | Key terms and corresponding MeSH terms | Number |
| 1 | "familial hypercholesterolaemia" OR "Hyperlipoproteinemia Type II"[Mesh] OR "Heterozygous Familial Hypercholesterolaemia" | 8147 |
| 2 | "Cost effectiveness" OR "Cost-Effectiveness Analysis"[Mesh] OR "Marginal Analysis"[Mesh] OR "Cost-Utility Analysis" OR "Cost- Benefits Analysis" OR "Benefits and Costs"[Mesh] OR "health economics" OR "economic evaluation" | 74571 |
| 3 | "screening" OR "Mass Screening"[Mesh] OR "Diagnosis"[Mesh] OR "Genetic Testing"[Mesh] | 9719318 |
| Sum | 1 AND 2 AND 3 | 47 |
|  |  |  |
| Web of Science | | |
|  | Key terms and corresponding MeSH terms | Number |
| 1 | ((TS=(familial hypercholesterolaemia)) AND TS=(Hyperlipoproteinemia Type II)) AND TS=(Heterozygous Familial Hypercholesterolaemia) | 9015 |
| 2 | (((((((((TS=(Cost effectiveness)) OR TS=(Cost-Effectiveness Analysis)) OR TS=(Costs and Cost Analysis)) OR TS=(economic evaluation)) OR TS=(Cost-Utility Analysis)) OR TS=(Costs and Benefits)) AND TS=(Marginal Analysis))) OR TS=(Benefits and Costs)) OR TS=(health economics) | 164831 |
| 3 | ((((TS=(screening)) OR TS=(Mass Screening))) OR TS=(Diagnostic Screening)) OR TS=(Genetic Testing) | 1583118 |
| Sum | 1 AND 2 AND 3 | 109 |
|  |  |  |
| Embase | | |
|  | Key terms and corresponding MeSH terms | Number |
| 1 | 'familial hypercholesterolaemia' OR 'heterozygous familial hypercholesterolaemia' OR (heterozygous AND familial AND ('hypercholesterolaemia'/exp OR hypercholesterolaemia)) | 2714 |
| 2 | 'cost effectiveness' OR 'economic AND evaluation' OR 'cost- AND benefits AND analysis' OR 'cost utility analysis' | 215019 |
| 3 | 'screening' OR 'mass AND screening' OR 'diagnosis' OR 'genetic AND testing' | 457137 |
| Sum | 1 AND 2 AND 3 | 33 |

| Science Direct | | |
| --- | --- | --- |
|  | Key terms and corresponding MeSH terms | Number |
| 1 | "familial hypercholesterolaemia" OR "Hyperlipoproteinemia Type II" OR "heterozygous familial hypercholesterolaemia" | 3611 |
| 2 | "Cost effectiveness" OR "Cost-Utility Analysis" OR "Costs and Benefits" OR "Economic Evaluation" OR "Health Economics" OR "Benefits and Costs" OR "Cost- Benefits Analysis" | 156769 |
| 3 | "Screening" OR "Mass Screening" OR "Diagnosis" OR "Genetic Testing" | 1000000+ |
| Sum | 1 AND 2 AND 3 | 103 |

| Health Technology Assessment Database | | |
| --- | --- | --- |
|  | Key terms and corresponding MeSH terms | Number |
| 1 | "Familial hypercholesterolaemia" OR "Hyperlipoproteinemia Type II" OR "Cardiomyopathy, Hypertrophic, Familial" | 21 |
| 2 | "Cost effectiveness" OR "Cost-Effectiveness Analysis" OR "Costs and Cost Analysis" OR "Economic Evaluation" OR "Cost-Utility Analysis" OR “Cost- Benefits Analysis” | 2650 |
| 3 | "Screening" OR "Mass Screening" OR "Diagnostic Screening Programs" OR "Genetic Testing" OR "Genetic Carrier Screening" | 2626 |
| Sum | 1 AND 2 AND 3 | 6 |

Table S2. Types of healthcare costs considered in the included articles.

| ID | Organization of screening | Consultation | Screening test | IC identification | Treatment | Follow up visits | Disease costs | Indirect costs |
| --- | --- | --- | --- | --- | --- | --- | --- | --- |
| Kerr et al. (2017) | × | × | √ | √ | √ | × | √ | × |
| Crosland et al. (2018) | √ | √ | √ | √ | √ | × | √ | × |
| McKay et al. (2018) | √ | √ | √ | √ | √ | √ | √ | × |
| Marks et al. (2002) | √ | √ | √ | √ | √ | √ | √ | × |
| Marks et al. (2003) | √ | √ | √ | × | √ | × | √ | × |
| Nherera et al. (2011) | √ | √ | √ | × | √ | × | √ | × |
| Ademi et al. (2020) | √ | × | √ | √ | √ | √ | √ | × |
| Ademi et al. (2014) | × | × | √ | √ | √ | √ | √ | × |
| Marquina et al. (2022) | √ | × | √ | √ | √ | √ | √ | √ |
| Chen et al. (2015) | √ | √ | √ | √ | √ | √ | √ | √ |
| Spencer et al. (2022) | √ | × | √ | √ | √ | × | √ | × |
| Jackson et al. (2021). | √ | √ | √ | × | √ | √ | × | × |
| Araujo et al. (2023) | × | √ | √ | √ | √ | √ | √ | × |
| Pelczarska et al.(2018) | √ | √ | √ | × | √ | √ | √ | × |
| Lázaro et al. (2017) | √ | × | √ | √ | √ | × | √ | √ |
| Oliva et al. (2009) | × | √ | √ | × | √ | √ | √ | × |
| Wonderling et al. (2004) | √ | √ | √ | √ | √ | √ | √ | × |
| Marang-van et al. (2002) | √ | √ | √ | √ | √ | √ | √ | × |
| Ademi et al. (2023) | √ | × | √ | √ | √ | √ | √ | √ |

√: Costs includes this.

×: This item is not included in the costs.

Table S3. Distribution of health and opportunity costs across diverse socioeconomic groups.

|  | IMD1 | IMD2 | IMD3 | IMD4 | IMD5 | Source |
| --- | --- | --- | --- | --- | --- | --- |
| Baseline distribution of health (QALE) | 63.21 | 67.61 | 69.95 | 73.10 | 75.00 | Love-Koh et al ^2^ |
| Health opportunity costs distribution | 26% | 22% | 22% | 16% | 14% | Love-Koh et al ^3^ |

Note: IMD, or Index of Multiple Deprivation, is a composite deprivation index used to measure health inequality. IMD1 refers to the most deprived areas, while IMD5 refers to the least deprived areas. Love-Koh et al.'s studies estimated the baseline distribution of health (quality-adjusted life expectancy, or QALE) and the distribution of health opportunity costs.

Table S4. Assessment of reporting quality by CHEERS (2022 version)

|  |  | Included study | | | | | | | | | | | | | | | | | | |
| --- | --- | --- | --- | --- | --- | --- | --- | --- | --- | --- | --- | --- | --- | --- | --- | --- | --- | --- | --- | --- |
| Reporting item | No. | 1 | 2 | 3 | 4 | 5 | 6 | 7 | 8 | 9 | 10 | 11 | 12 | 13 | 14 | 15 | 16 | 17 | 18 | 19 |
| Sum points | | 22.5 | 22 | 21 | 20 | 19 | 22 | 23.5 | 20.5 | 24.5 | 21 | 22.5 | 21.5 | 21 | 21 | 21 | 21.5 | 20 | 20.5 | 21.5 |
| **TITLE** | | | | | | | | | | | | | | | | | | | | |
| Title | 1 | 1 | 1 | 1 | 0.5 | 0.5 | 1 | 0.5 | 1 | 1 | 0.5 | 1 | 1 | 1 | 0.5 | 1 | 0.5 | 0.5 | 0.5 | 0.5 |
| **ABSTRACT** | | | | | | | | | | | | | | | | | | | | |
| Abstract | 2 | 1 | 1 | 1 | 1 | 1 | 1 | 1 | 1 | 1 | 1 | 1 | 1 | 1 | 1 | 1 | 1 | 0.5 | 1 | 1 |
| **INTRODUCTION** | | | | | | | | | | | | | | | | | | | | |
| Background and objectives | 3 | 1 | 1 | 1 | 1 | 1 | 1 | 1 | 1 | 1 | 1 | 1 | 1 | 1 | 1 | 1 | 1 | 1 | 1 | 1 |
| **METHODS** | | | | | | | | | | | | | | | | | | | | |
| Health economic analysis plan | 4 | 1 | 1 | 1 | 1 | 1 | 1 | 1 | 1 | 1 | 1 | 1 | 1 | 1 | 1 | 1 | 1 | 1 | 1 | 1 |
| Study population | 5 | 1 | 1 | 1 | 1 | 1 | 1 | 1 | 1 | 1 | 1 | 0.5 | 1 | 0.5 | 1 | 1 | 1 | 0.5 | 1 | 1 |
| Setting and location | 6 | 1 | 1 | 1 | 1 | 1 | 1 | 1 | 1 | 1 | 1 | 1 | 1 | 1 | 1 | 1 | 1 | 1 | 1 | 1 |
| Comparators | 7 | 1 | 1 | 1 | 1 | 1 | 1 | 1 | 1 | 1 | 1 | 1 | 1 | 1 | 1 | 1 | 1 | 1 | 1 | 1 |
| Perspective | 8 | 1 | 1 | 1 | 0.5 | 0.5 | 1 | 1 | 1 | 1 | 1 | 1 | 1 | 1 | 1 | 1 | 1 | 0.5 | 0.5 | 1 |
| Time horizon | 9 | 0.5 | 0.5 | 0.5 | 0.5 | 0.5 | 0.5 | 0.5 | 0.5 | 0.5 | 0.5 | 0.5 | 0.5 | 0.5 | 0.5 | 0.5 | 0.5 | 0.5 | 1 | 0.5 |
| Discount rate | 10 | 0.5 | 1 | 0.5 | 1 | 0 | 1 | 1 | 0.5 | 1 | 0.5 | 0.5 | 0.5 | 0.5 | 1 | 0.5 | 0.5 | 1 | 0.5 | 1 |
| Selection of outcomes | 11 | 1 | 1 | 1 | 1 | 1 | 1 | 1 | 1 | 1 | 1 | 1 | 1 | 1 | 1 | 1 | 1 | 1 | 1 | 1 |
| Measurement of outcomes | 12 | 1 | 1 | 1 | 1 | 1 | 1 | 1 | 1 | 1 | 1 | 1 | 1 | 1 | 1 | 1 | 1 | 1 | 1 | 1 |
| Valuation of outcomes | 13 | 1 | 1 | 1 | 1 | 1 | 1 | 1 | 1 | 1 | 1 | 1 | 1 | 1 | 1 | 1 | 1 | 1 | 1 | 1 |
| Measurement and valuation of resources and costs | 14 | 1 | 1 | 1 | 1 | 1 | 1 | 1 | 1 | 1 | 1 | 1 | 1 | 1 | 1 | 1 | 1 | 1 | 1 | 1 |
| Currency, price date, and conversion | 15 | 1 | 1 | 1 | 0 | 0 | 1 | 1 | 1 | 1 | 1 | 1 | 1 | 0.5 | 0.5 | 1 | 1 | 1 | 1 | 1 |
| Rationale and description of model | 16 | 0.5 | 1 | 0.5 | 0.5 | 0.5 | 0.5 | 0.5 | 0.5 | 0.5 | 0.5 | 0.5 | 1 | 1 | 0.5 | 0.5 | 0.5 | 0.5 | 0.5 | 0.5 |
| Analytics and assumptions | 17 | 0.5 | 0.5 | 0.5 | 0 | 0.5 | 0.5 | 0.5 | 0.5 | 1 | 0.5 | 1 | 1 | 0 | 0.5 | 0.5 | 0.5 | 0.5 | 1 | 0.5 |
| Characterizing heterogeneity | 18 | 0 | 0 | 0 | 0 | 0 | 0 | 1 | 0 | 1 | 0 | 0 | 0 | 0 | 0 | 0 | 0 | 0 | 0 | 0 |
| Characterizing distributional effects | 19 | 1 | 0 | 0 | 1 | 1 | 0 | 1 | 0 | 1 | 0 | 1 | 1 | 1 | 0 | 0 | 1 | 1 | 1 | 1 |
| Characterizing uncertainty | 20 | 0.5 | 0.5 | 0.5 | 0.5 | 0.5 | 0.5 | 0.5 | 0.5 | 0.5 | 0.5 | 0.5 | 0 | 0.5 | 0.5 | 0.5 | 0.5 | 0.5 | 0.5 | 0.5 |
| Approach to engagement with patients and others affected by the study | 21 | 0.5 | 0.5 | 0.5 | 0.5 | 0.5 | 0.5 | 0.5 | 0.5 | 0.5 | 0.5 | 0.5 | 0.5 | 0.5 | 0.5 | 0.5 | 0.5 | 0.5 | 0.5 | 0.5 |
| **RESULTS** | | | | | | | | | | | | | | | | | | | | |
| Study parameters | 22 | 1 | 1 | 1 | 1 | 1 | 1 | 1 | 1 | 1 | 1 | 1 | 0.5 | 1 | 1 | 1 | 1 | 1 | 1 | 1 |
| Summary of main results | 23 | 1 | 0.5 | 0.5 | 0.5 | 1 | 1 | 0.5 | 1 | 1 | 1 | 1 | 0.5 | 0.5 | 1 | 1 | 1 | 1 | 1 | 1 |
| Effect of uncertainty | 24 | 0.5 | 0.5 | 0.5 | 0.5 | 0.5 | 0.5 | 1 | 0.5 | 0.5 | 0.5 | 0.5 | 0 | 0.5 | 0.5 | 1 | 1 | 0.5 | 0.5 | 0.5 |
| Effect of engagement with patients and others affected by the study | 25 | 0 | 0 | 0 | 0 | 0 | 0 | 0 | 0 | 0 | 0 | 0 | 0 | 0 | 0 | 0 | 0 | 0 | 0 | 0 |
| **DISCUSSION** | | | | | | | | | | | | | | | | | | | | |
| Study findings, limitations, generalizability, and current knowledge | 26 | 1 | 1 | 1 | 1 | 1 | 1 | 1 | 1 | 1 | 1 | 1 | 1 | 1 | 1 | 1 | 1 | 1 | 1 | 1 |
| Source of funding | 27 | 1 | 1 | 1 | 1 | 1 | 1 | 1 | 1 | 1 | 1 | 1 | 1 | 1 | 1 | 1 | 1 | 1 | 0 | 0 |
| Conflicts of interest | 28 | 1 | 1 | 1 | 1 | 0 | 1 | 1 | 0 | 1 | 1 | 1 | 1 | 1 | 1 | 1 | 0 | 0 | 0 | 1 |

Note: Green: fully satisfied. Light green: Partially satisfied. Red: Not satisfied. Study ID: 1, Kerr2017; 2, Crosland,2018; 3, McKay,2018; 4, Marks,2002; 5, Marks,2003; 6, Nherera ,2011; 7, Ademi, 2020; 8, Ademi,2014; 9, Marquina, 2022; 10, Chen,2015; 11, Spencer,2022; 12, Jackson,2021; 13, Araujo,2023; 14, Pelczarska ,2018; 15, Lázaro,2017; 16, Oliva,2009; 17, Wonderling,2004; 18; Marang-van 2002; 19, Ademi,2023.

Table S5. Quality assessment by QHES.

| Item | Points | Included study | | | | | | | | | | | | | | | | | | |
| --- | --- | --- | --- | --- | --- | --- | --- | --- | --- | --- | --- | --- | --- | --- | --- | --- | --- | --- | --- | --- |
|  |  | 1 | 2 | 3 | 4 | 5 | 6 | 7 | 8 | 9 | 10 | 11 | 12 | 13 | 14 | 15 | 16 | 17 | 18 | 19 |
| 1 | 7 | 7 | 7 | 7 | 7 | 7 | 7 | 7 | 7 | 7 | 7 | 7 | 7 | 7 | 7 | 7 | 7 | 7 | 7 | 7 |
| 2 | 4 | 4 | 4 | 4 | 2 | 2 | 4 | 4 | 4 | 4 | 4 | 4 | 4 | 4 | 4 | 4 | 4 | 2 | 2 | 4 |
| 3 | 8 | 7 | 7 | 7 | 7 | 6 | 6 | 7 | 6 | 7 | 6 | 6 | 6 | 6 | 7 | 7 | 6 | 6 | 7 | 7 |
| 4 | 1 | 0 | 0 | 0 | 0 | 0 | 0 | 1 | 0 | 1 | 0 | 0 | 0 | 0 | 0 | 0 | 0 | 0 | 0 | 0 |
| 5 | 9 | 7 | 9 | 9 | 7 | 5 | 7 | 7 | 7 | 7 | 7 | 7 | 1 | 7 | 7 | 7 | 9 | 5 | 6 | 9 |
| 6 | 6 | 6 | 3 | 3 | 3 | 6 | 6 | 6 | 6 | 6 | 6 | 6 | 3 | 3 | 6 | 6 | 6 | 6 | 3 | 6 |
| 7 | 5 | 5 | 5 | 5 | 5 | 5 | 5 | 5 | 5 | 5 | 5 | 5 | 5 | 5 | 5 | 5 | 5 | 5 | 5 | 5 |
| 8 | 7 | 5 | 7 | 5 | 7 | 4 | 7 | 7 | 5 | 7 | 5 | 5 | 5 | 5 | 7 | 5 | 5 | 7 | 5 | 7 |
| 9 | 8 | 8 | 8 | 8 | 6 | 6 | 8 | 8 | 8 | 8 | 8 | 8 | 8 | 7 | 7 | 8 | 8 | 8 | 8 | 8 |
| 10 | 6 | 6 | 6 | 6 | 6 | 6 | 6 | 6 | 6 | 6 | 6 | 6 | 6 | 6 | 6 | 6 | 6 | 6 | 6 | 6 |
| 11 | 7 | 7 | 7 | 7 | 7 | 7 | 7 | 7 | 7 | 7 | 7 | 7 | 7 | 7 | 7 | 7 | 7 | 7 | 7 | 7 |
| 12 | 8 | 7 | 8 | 7 | 7 | 7 | 7 | 7 | 7 | 7 | 7 | 7 | 8 | 8 | 7 | 7 | 7 | 7 | 7 | 7 |
| 13 | 7 | 3 | 5 | 3 | 4 | 4 | 4 | 4 | 5 | 4 | 5 | 5 | 4 | 5 | 4 | 4 | 4 | 4 | 5 | 4 |
| 14 | 6 | 4 | 4 | 4 | 3 | 4 | 4 | 4 | 4 | 6 | 4 | 6 | 6 | 3 | 4 | 4 | 4 | 4 | 6 | 4 |
| 15 | 8 | 8 | 8 | 8 | 8 | 8 | 8 | 8 | 8 | 8 | 8 | 8 | 8 | 8 | 8 | 8 | 8 | 8 | 8 | 8 |
| 16 | 3 | 3 | 3 | 3 | 3 | 3 | 3 | 3 | 3 | 3 | 3 | 3 | 3 | 3 | 3 | 3 | 3 | 3 | 0 | 0 |
| SUM | 100 | 87 | 91 | 86 | 82 | 80 | 89 | 91 | 88 | 93 | 88 | 90 | 81 | 84 | 89 | 88 | 89 | 85 | 82 | 89 |

Note: 75-100: high quality; 50-74：moderate quality; 25-49: low quality; Study ID: 1, Kerr2017; 2, Crosland,2018; 3, McKay,2018; 4, Marks,2002; 5, Marks,2003; 6, Nherera ,2011; 7, Ademi, 2020; 8, Ademi,2014; 9, Marquina, 2022; 10, Chen,2015; 11, Spencer,2022; 12, Jackson,2021; 13, Araujo,2023; 14, Pelczarska ,2018; 15, Lázaro,2017; 16, Oliva,2009; 17, Wonderling,2004; 18; Marang-van 2002; 19, Ademi,2023.

Table S6. Data extraction for cascade screening (costs converted to 2023 US dollars)

| Author  Year | Incremental cost | QALY gain | LYG | Total advents averted | Adverse events averted | Deaths averted |
| --- | --- | --- | --- | --- | --- | --- |
| Kerr et al. (2017) | 4702.79 | 0.48 | NA | 46 MIs,50 cases of angina,8 strokes and 16 deaths | 104 | 16 |
| Marks et al. (2003) | 101486932.5 | NA | NA | 560 deaths |  | 560 |
| Ademi et al. (2020) | -852.31 | 1.07 | 0.97 | 24.2 fewer acute non-fatal events and 7.55 death | 24.2 | 7.55 |
| Ademi et al. (2014) | 84620.28 | 29.07 | 24.95 | NA | NA | NA |
| Lázaro et al. (2017) | 39591894.47 | 767 | NA | 847Cardiac events;203Coronary deaths | 847 | 203 |
| Oliva et al. (2009) | 8922.69 | NA | 1.34 | NA | NA | NA |
| Wonderling et al. (2004) | 11433.06 | NA | 0.9 | NA | NA | NA |
| Ademi et al. (2023) | 31369.18 | 2.53 | 2.28 | NA | NA | NA |

Note: Green: Reported QALYs; Yellow: Reported LYGs; Pink: Total CE; Grey: Reported adverse events averted; Blue: Reported deaths averted.

Table S7. Data extraction for universal screening (costs converted to 2023 US dollars).

| Author  Year | Incremental cost | QALY gain | LYG | Total advents averted | Adverse events averted | Deaths averted |
| --- | --- | --- | --- | --- | --- | --- |
| Marks et al. (2003) | 13500753.09 | NA | NA | 11.7 deaths |  | 11.7 |
| Marquina et al. (2022) | 1061544599.77 | 51790 | 33488 | 3093 CHD events | 1813 | 1279 |
| Spencer et al. (2022) | 20836899.42 | 97.5 | 61.5 | NA | NA | NA |

Note: Green: Reported QALYs; Yellow: Reported LYGs; Pink: Total CE; Grey: Reported adverse events averted; Blue: Reported deaths averted.

Table S8. PRISMA checklist.

| **Section and**  **Topic** | **Item #** | **Checklist item** | **Location**  **where item is reported** |
| --- | --- | --- | --- |
| **TITLE** | | |  |
| Title | 1 | Identify the report as a systematic review. | 1 |
| **ABSTRACT** | | |  |
| Abstract | 2 | See the PRISMA 2020 for Abstracts checklist. | 2-3 |
| **INTRODUCTION** | | |  |
| Rationale | 3 | Describe the rationale for the review in the context of existing knowledge. | 4-5 |
| Objectives | 4 | Provide an explicit statement of the objective(s) or question(s) the review addresses. | 6 |
| **METHODS** | | |  |
| Eligibility criteria | 5 | Specify the inclusion and exclusion criteria for the review and how studies were grouped for the syntheses. | 7-8 |
| Information  sources | 6 | Specify all databases, registers, websites, organisations, reference lists and other sources searched or consulted to identify studies. Specify the date when each source was last searched or consulted. | 7 |
| Search strategy | 7 | Present the full search strategies for all databases, registers and websites, including any filters and limits used. | 8 |
| Selection process | 8 | Specify the methods used to decide whether a study met the inclusion criteria of the review, including how many reviewers screened each record and each report retrieved, whether they worked independently, and if applicable, details of automation tools used in the process. | 7 |
| Data collection  process | 9 | Specify the methods used to collect data from reports, including how many reviewers collected data from each report, whether they worked  independently, any processes for obtaining or confirming data from study investigators, and if applicable, details of automation tools used in the process. | 8 |
| Data items | 10a | List and define all outcomes for which data were sought. Specify whether all results that were compatible with each outcome domain in each study were sought (e.g. for all measures, time points, analyses), and if not, the methods used to decide which results to collect. | 8 |
|  | 10b | List and define all other variables for which data were sought (e.g. participant and intervention characteristics, funding sources). Describe any assumptions made about any missing or unclear information. | 8-9 |
| Study risk of bias assessment | 11 | Specify the methods used to assess risk of bias in the included studies, including details of the tool(s) used, how many reviewers assessed each study and whether they worked independently, and if applicable, details of automation tools used in the process. | 8-9 |
| Effect measures | 12 | Specify for each outcome the effect measure(s) (e.g. risk ratio, mean difference) used in the synthesis or presentation of results. | 10-11 |
| Synthesis  methods | 13a | Describe the processes used to decide which studies were eligible for each synthesis (e.g. tabulating the study intervention characteristics and comparing against the planned groups for each synthesis (item #5)). | 10-11  35-43 |
|  | 13b | Describe any methods required to prepare the data for presentation or synthesis, such as handling of missing summary statistics, or data conversions. | 11 |
|  | 13c | Describe any methods used to tabulate or visually display results of individual studies and syntheses. | 10-11 |
|  | 13d | Describe any methods used to synthesize results and provide a rationale for the choice(s). If meta-analysis was performed, describe the model(s), method(s) to identify the presence and extent of statistical heterogeneity, and software package(s) used. | 10-11 |
|  | 13e | Describe any methods used to explore possible causes of heterogeneity among study results (e.g. subgroup analysis, meta-regression). | 9-11 |
|  | 13f | Describe any sensitivity analyses conducted to assess robustness of the synthesized results. |  |
| Reporting bias  assessment | 14 | Describe any methods used to assess risk of bias due to missing results in a synthesis (arising from reporting biases). |  |
| Certainty  assessment | 15 | Describe any methods used to assess certainty (or confidence) in the body of evidence for an outcome. | 11 |
| **RESULTS** | | |  |
| Study selection | 16a | Describe the results of the search and selection process, from the number of records identified in the search to the number of studies included in the review, ideally using a flow diagram. | 16  44 |
|  | 16b | Cite studies that might appear to meet the inclusion criteria, but which were excluded, and explain why they were excluded. | 16 |
| Study  characteristics | 17 | Cite each included study and present its characteristics. | 17-20 |
| Risk of bias in  studies | 18 | Present assessments of risk of bias for each included study. | 16 |
| Results of  individual studies | 19 | For all outcomes, present, for each study: (a) summary statistics for each group (where appropriate) and (b) an effect estimate and its precision (e.g. confidence/credible interval), ideally using structured tables or plots. | 35-43 |
| Results of  syntheses | 20a | For each synthesis, briefly summarise the characteristics and risk of bias among contributing studies. | 17-20 |
|  | 20b | Present results of all statistical syntheses conducted. If meta-analysis was done, present for each the summary estimate and its precision (e.g. confidence/credible interval) and measures of statistical heterogeneity. If comparing groups, describe the direction of the effect. | 21-22 |
|  | 20c | Present results of all investigations of possible causes of heterogeneity among study results. |  |
|  | 20d | Present results of all sensitivity analyses conducted to assess the robustness of the synthesized results. |  |
| Reporting biases | 21 | Present assessments of risk of bias due to missing results (arising from reporting biases) for each synthesis assessed. |  |
| Certainty of  evidence | 22 | Present assessments of certainty (or confidence) in the body of evidence for each outcome assessed. | 21-22  43 |
| **DISCUSSION** | | |  |
| Discussion | 23a | Provide a general interpretation of the results in the context of other evidence. | 26 |
|  | 23b | Discuss any limitations of the evidence included in the review. |  |
|  | 23c | Discuss any limitations of the review processes used. |  |
|  | 23d | Discuss implications of the results for practice, policy, and future research. | 27-29 |
| **OTHER INFORMATION** | | |  |
| Registration and protocol | 24a | Provide registration information for the review, including register name and registration number, or state that the review was not registered. |  |
|  | 24b | Indicate where the review protocol can be accessed, or state that a protocol was not prepared. |  |
|  | 24c | Describe and explain any amendments to information provided at registration or in the protocol. |  |
| Support | 25 | Describe sources of financial or non-financial support for the review, and the role of the funders or sponsors in the review. |  |
| Competing  interests | 26 | Declare any competing interests of review authors. | 1 |
| Availability of  data, code and  other materials | 27 | Report which of the following are publicly available and where they can be found: template data collection forms; data extracted from included studies; data used for all analyses; analytic code; any other materials used in the review. |  |

Note: source: Page MJ, McKenzie JE, Bossuyt PM, Boutron I, Hoffmann TC, Mulrow CD, et al. The PRISMA 2020 statement: an updated guideline for reporting systematic reviews. BMJ 2021;372:n71. doi: 10.1136/bmj.n71

# References

1. Crespo C, Monleon A, Díaz W, Ríos M. Comparative efficiency research (COMER): meta-analysis of cost-effectiveness studies. *BMC Medical Research Methodology* 2014; **14**(1): 139.

2. Love-Koh J, Asaria M, Cookson R, Griffin S. The Social Distribution of Health: Estimating Quality-Adjusted Life Expectancy in England. *Value in Health* 2015; **18**(5): 655-62.

3. Love-Koh J, Cookson R, Claxton K, Griffin S. Estimating Social Variation in the Health Effects of Changes in Health Care Expenditure. *Medical Decision Making* 2020; **40**(2): 170-82.
